# Public Opinions on COVID-19 Vaccines—A Spatiotemporal Perspective on Races and Topics Using a Bayesian-Based Method

**DOI:** 10.3390/vaccines10091486

**Published:** 2022-09-07

**Authors:** Zifu Wang, Yudi Chen, Yun Li, Devika Kakkar, Wendy Guan, Wenying Ji, Jacob Cain, Hai Lan, Dexuan Sha, Qian Liu, Chaowei Yang

**Affiliations:** 1 Department of Geography and Geoinformation Science, NSF Spatiotemporal Innovation Center, George Mason University, Fairfax, VA 22030, USA; 2 Department of Civil, Environmental and Infrastructure Engineering, George Mason University, Fairfax, VA 22030, USA; 3 Center for Geographic Analysis, Harvard University, Cambridge, MA 02138, USA; 4 Department of Geography, Frostburg State University, Frostburg, MD 21532, USA

**Keywords:** social media, public opinions, COVID-19 vaccines, spatiotemporal analysis, race inequality, bayesian inference

## Abstract

The COVID-19 pandemic has been sweeping across the United States of America since early 2020. The whole world was waiting for vaccination to end this pandemic. Since the approval of the first vaccine by the U.S. CDC on 9 November 2020, nearly 67.5% of the US population have been fully vaccinated by 10 July 2022. While quite successful in controlling the spreading of COVID-19, there were voices against vaccines. Therefore, this research utilizes geo-tweets and Bayesian-based method to investigate public opinions towards vaccines based on (1) the spatiotemporal changes in public engagement and public sentiment; (2) how the public engagement and sentiment react to different vaccine-related topics; (3) how various races behave differently. We connected the phenomenon observed to real-time and historical events. We found that in general the public is positive towards COVID-19 vaccines. Public sentiment positivity went up as more people were vaccinated. Public sentiment on specific topics varied in different periods. African Americans’ sentiment toward vaccines was relatively lower than other races.

## 1. Introduction

In December 2019, the first occurrence of severe acute respiratory syndrome coronavirus which came to be known as COVID-19 was reported in Wuhan, China. COVID-19 quickly spread within the city and to other countries, due to its notably high transmission rate. The first case in the US was documented in late January 2020 and by mid-March, infection cases had been reported in all 50 states and the US had the most documented COVID-19 death cases in the world by mid-April 2020 [1]. As of 17 May 2022, more than 83 million cases and over 1 million deaths were confirmed in the United States [2]. Unlike the regular flu virus, the susceptibility and lethality of COVID-19 have alerted people to take strict precautions. To combat the pandemic, US federal and state government agencies implemented a series of social distancing and lockdown measures, e.g., school closure, workplace closures, stay-at-home orders, and travel controls [3,4], which have been argued to be some of the effective means to combat the spreading of COVID-19 before a vaccine becomes available [5]. However, these orders coincided with high unemployment rates and economic downturns. Due to the high transmissibility of COVID-19 and its variants, as well as the negative economic, social, and medical implications of this pandemic, controlling COVID-19′s spread has become direr than ever. The number of daily confirmed cases rose to the highest point on 18 January 2022; more than one million new cases were found that day [1].

As containment and closure measures are not always economically sustainable nor easy to follow, vaccines are useful for fighting COVID-19 by achieving herd immunity. Many companies in the US started developing COVID-19 vaccines in March 2020 [6]. In December, the Food and Drug Administration (FDA) authorized the emergency use of both the Pfizer-BioNTech and Moderna vaccines. Although these vaccines are asserted to be effective, research and vaccine development is still ongoing as there are still many unanswered questions. For example, some doctors think that some populations may respond better to certain vaccine types [7]. Additionally, the initial vaccine trials included participants who were 16 and older. Vaccine recommendations for children remain undetermined until 17 June 2022, when the FDA authorized Moderna and Pfizer-BioNTech COVID-19 Vaccines for children as young as 6 months of age [8]. It was also uncertain whether the vaccine prevents asymptomatic cases and their spreading, or just severe cases. These uncertainties pose grand challenges to the vaccination progress in the US. However, a 70–80% vaccination rate is needed for achieving herd immunity.

There remain many difficulties associated with vaccination. For example, before the COVID-19 pandemic started, a study shows hesitant attitudes to vaccination are prevalent and may be increasing since the influenza pandemic of 2009. During the COVID-19 pandemic [9], people who protested early pandemic mitigation measures, such as stay-at-home orders and mask mandates, are likewise protesting mandatory vaccines. Some are resisting mandatory vaccines until detailed data from the trials is made publicly available [10]. Others oppose the vaccine due to false information that has proliferated on the internet—such as claims of the vaccine killing people, and implanting microchips, among other conspiracies [11]. Lastly, the suspended use of the AstraZeneca COVID-19 vaccine did bring concerns to the worldwide public [12]. Therefore, understanding the public’s attitude towards vaccines from different perspectives is necessary for public health administrators to design communication strategies targeting different populations, such as by regions and races, to convince them to accept vaccines, which can help control COVID-19 cases from spreading.

To reach the 70–80% vaccination rate needed for herd immunity, public health administrators are suggested to focus on public attitude and education rather than making vaccines mandatory [4,10]. Questionnaires could be a useful way to understand the public’s attitude towards vaccines. However, the coverage of questionnaires is limited, meanwhile, it is costly and labor intensive. Alternatively, with the growing popularity of social media, an increasing number of people post blogs about their opinions regarding social/natural events on social media platforms, which provides near-real time and valuable information for mining and analyzing public attitudes towards spatiotemporal events. Thus, this study leveraged social media data to investigate (1) how public engagement and the public sentiment toward vaccines change spatiotemporally; (2) how the public reacts toward different topics related to vaccines; (3) how various races behave differently toward vaccines.

## 2. Literature Review

### 2.1. Using Social Media to Analyze Opinions for Public Events

Conducting surveys is a primary method to capture mass public opinions [13], but it has not gone without criticism. Using surveys to reveal public opinion often neglects the social aspects of public opinion [14]. People who took the surveys are defined as the number of people holding a certain opinion and the people holding that opinion would be identified as belonging to the public [15]. In addition, survey results are built on the assumption that people honestly expressed their genuine opinions in a public environment [14]. Finally, contemporary methods of conducting surveys may not be sufficient as they usually restrict the ability to capture the spatiotemporal dynamics of their survey objects [16]. Public sentiment toward social events, such as COVID-19 vaccination, often varies spatiotemporally with different perspectives of the incidents [17].

Social media compensates for the disadvantages of surveys outlined above. First, data from social media is not sampled representatively by regions, ages, genders, education levels, social classes, and other metrics. The analysis based on social media collects all available data such as content, likes, shares, and comments, which reveal the opinion of the engaging public [14]. In addition, users can anonymously create social media accounts and post their opinions without taking accountability for disrespectful posts. Social media creates a crowd-sourced and near a real-time information source, and has the potential to reveal the dynamics of human activities by allowing for processing of the public posts, such as for natural disaster events, which are often updated every minute [18]. Lastly, information posted on social media is publicly available and can be easily collected. All these characteristics make social media promising to be widely used in analyzing social events to understand public opinions. This study utilizes Twitter as the social media data source to investigate public sentiment.

Public engagement level is an important measure of public opinion [19] as it values the degree of the public willingness to discuss certain issues. Prior studies had quantified various methods of measuring public engagement. Researchers had utilized social media built-in features, such as the number of comments, retweets, and likes for Twitter [20], or had built their own metrics, such as the ratio of total likes to total posts [21]. Our study needs to investigate public engagement in certain geographic regions, but social media activities in a geographic region or a demographic group are highly affected by its population size. A previous study pointed out that population density can predict how many users and tweets we should see in an area [22]. In other words, regions with high population densities are correlated with a greater number of social media posts. In addition, potential bias may also exist in social media due to different races and regions [23]. Therefore, our study proposes an engagement score measurement, which normalizes the population differences to achieve a reliable comparison of public engagement in social media among different geographic regions and demographic groups.

In recent years, social media has also played a vital role in measuring public sentiment to understand reactions toward events [24]. The sentiment value of a social media post is typically classified into three sentiment polarities (i.e., positive, neutral, and negative) [25], and then a public sentiment value is derived by aggregating and averaging the sentiment values of all posts. Many studies in recent years utilized models and tools which implemented probability distribution to analyze the text sentiment, such as the LDA Model and Vader [26,27], which utilized Textblob with Naïve Bayes categorization model which also implemented probability distribution concepts to determine the sentiment value of tweets. Public sentiment values cannot represent the sentiment of the entire user population on social media platforms because the tweets sampled reflect only a small portion of the entire population of social media users [28,29]. Therefore, such a public sentiment value can only be a probability value with uncertainties. The more posts on a social media platform, the more certain the probability is and vice versa [30]. Therefore, this study implements a Bayesian inference approach proposed by Chen [28] to reliably estimate the sentiment uncertainty.

### 2.2. Public Opinions toward COVID-19 Vaccinations

A vaccine is a substance used to stimulate the production of antibodies and provide immunity against one or several diseases [31], but strong sentiment against vaccination exists among the global population. Previous studies have found various motivations which drive anti-vaccine sentiment, including suspected side effects caused by vaccines (e.g., autism), distrust of government, and unreliable development processes [32]. Despite the above implications being scientifically debunked, such as the unfound correlation between vaccinations and autism, some of the arguments are indeed due to historical reasons and the consequences of reality. For example, in 1932, the U.S. Public Health Service (USPHS), working with the Tuskegee Institute, began a study to record the natural history of syphilis. Six hundred African Americans had been involved without their informed consent for 40 years [33]. Such abuse has reduced the trust in the government among African Americans [34]. In addition, people had questioned the maturity of vaccines, especially regarding the COVID-19 vaccines. Vaccine development is a long and complex process, which often lasts 10 to 15 years. However, the development time of the three approved vaccines (Pfizer-BioNTech, Moderna, and Johnson & Johnson) in the U.S. only lasted around 1 year, which is much shorter than usual. Previous studies have investigated public opinions toward COVID-19 vaccines by utilizing surveys. These surveys contained comprehensive questions with specific metrics and were distributed to representative groups comprised of different ages, genders, professions, and geo-locations [35,36,37]. However, this research demonstrated different results of public sentiment toward receiving COVID-19 vaccination from their surveys and the results from such research are not representative of the public. In addition, prior studies have also utilized social media to investigate public opinion toward COVID-19 vaccines in 2020 [38,39], but the first vaccine was approved by the FDA (U.S. Food and Drug Administration) on 9 November 2020 [40], and the first dose was administered on 14 December [41]. After the public administration of COVID-19 vaccines, the spatiotemporal patterns of public sentiment toward such vaccines remain little-known. Due to the lack of research to understand public opinions toward the COVID-19 vaccine by social media in the United States, our study is proposed to investigate the public engagement and sentiment toward vaccines and compare the spatiotemporal patterns of both measures before and after the availability of vaccines.

Finally, existing studies have shown different treatments in clinical trials and the effects of vaccines for different races. In the clinical trials of two major brands of vaccines (Pfizer/BioNTech, and Moderna) approved by the U.S. CDC, the number of white participants is always the highest (Pfizer/BioNTech 81.9%, Moderna 79.4%) [42]. Whether the vaccines are effective for all minorities is of great concern for minority groups. Therefore, understanding public opinions of different races are also necessary, and this study investigates public engagement and sentiment of all races towards vaccines.

### 2.3. Previous Studies on Public Opinion towards COVID-19 Vaccine

There are some existing studies on public opinions toward COVID-19 vaccines. However, the existing studies have certain shortcomings in the following three aspects. First, the research period is too short to cover all perspectives of COVID-19 vaccines [43,44]. The existing studies only cover their social media data from January 2020 up to January 2021. However, at that time only around 2.5% of the population in the United States received at least one dose as most of the public was still waiting to receive the vaccines [45]. Many perspectives cannot be fully analyzed. For example, sentiment on vaccine effectiveness cannot be determined because most people were not vaccinated, and therefore their feelings after receiving vaccination were not studied. This study extends the study period to May 2021, which is the time more than 50% of the population received at least one dose [45].

In addition, one social media post can express multiple distinct types of sentiment regarding to different perspectives. For example, a person is positive about receiving the vaccine meanwhile complaining about the slow administration process of receiving the vaccine. To observe what vaccine-related topics have been discussed and how the sentiment varies toward different aspects of vaccines, three perspectives are defined and investigated separately in this research.

Lastly, different races have a different attitude and different vaccination rates toward COVID-19 vaccines. The existing studies have not revealed the social behavior of different races on social media. A survey that investigated racial and ethnic differences in COVID-19 vaccine hesitancy among health care workers (HCW) showed that vaccine hesitancy was nearly 5-fold higher among Black HCWs compared with White counterparts, 2-fold higher among Hispanic or Latino HCWs, and close to 50% lower among Asian HCWs and HCWs who were members of other racial/ethnic groups [46]. This study investigated different races on social media to demonstrate the differences in their public opinions on the COVID-19 vaccine.

## 3. Data

### 3.1. Social Media Data

Twitter provides large-scale and easy-access social media datasets for social media analysis. This study harvested Geo-tagged Tweet data by searching keywords “vaccine” and “vaccination” from the Harvard Center for Geographic Analysis (CGA) Geotweets Archive [47]. The Harvard Center for Geographic Analysis (CGA) maintains the Geotweet Archive, a global record of tweets spanning time, geography, and language. The primary purpose of the Archive is to make a comprehensive collection of geo-located tweets available to the academic research community. The archive extends from 2010 to the present and is updated daily and the number of tweets in the collection totals approximately 10 billion. The data is collected using Twitter’s Streaming API following Twitter’s Developer Agreement and Policy [47].

The Geotweet Archive consists of tweets that carry two types of geospatial signature: (1) GPS-based longitude/latitude generated by the originating device. (2) Place-name-centroid-based longitude/latitude from the bounding box provided by Twitter, based on the user-defined place designation [47].

Any tweet which carries one or both signatures is included in the Archive. Approximately 1–2% of all tweets contain such geographic coordinates. The current version of the Archive is Version 2.0. The original Version 1.0 archive began in 2012 as part of a project to develop a GPU-powered spatial database called GEOPS. Version 2.0 of the archive represents the results of a merge between the CGA archive, and an archive developed by the Department of Geoinformatics at the University of Salzburg in Austria, as well as several other archives [47].

For the purposes of ethical approval, this study removed specific twitter identities for protecting the privacy of users after generating statistical results. Only statistical results are presented in this article.

The data excluded retweets and were all English-speaking tweets in the contiguous United States which cover 48 adjoining states and the District of Columbia. The dataset ranged from 1 October 2020 to 21 May 2021. 156,207 tweets have been collected for this study. The attributes message_id, tweet_date, tweet_text, tags, user_id, user_name, user_location, latitude, and longitude were used for analysis in this research.

### 3.2. States Demographics

To fairly compare the engagement level among different states, the tweet quantity of a given state was normalized by dividing the population size of that respective state. The latest total population estimation data and the race distribution data in each state and the whole nation were collected from US Census Bureau [48]. The attributes, including state name, population size, and ratio for each race were derived from these datasets in this study.

### 3.3. Race Distribution for the Last Names

This study also investigated the engagement and sentiment levels toward vaccines from different races so that public opinion toward vaccines among different races can be better understood. To obtain the race of each tweet, this study matched the last name of the user from each tweet to the last-name database released by the U.S. Census Bureau [48]. The U.S. Census Bureau tabulates the percentage distribution of race/ethnicity for all last names occurring 100 or more times in 2010. The races are classified as White, Black, API (Asian and Pacific Islander), AIAN (American Indian and Alaska Native), 2Prace (more than two races), and Hispanics [48]. As an example, in Table 1, the last name “Washington” is observed to correspond to White 5.17%, Black 87.53%, API 0.3%, AIAN 0.68%, 2Prace 3.78%, and Hispanic 2.54%. It means that in the United States, 87.53% of the population with the last name “Washington” are African Americans.

### 3.4. COVID-19 Case Data

The number of COVID-19 cases is an important indicator that affects public engagement and sentiment toward vaccines. To investigate the relationship between COVID-19 cases and public engagement and sentiment to vaccines, COVID-19 daily-confirmed-case data were collected in support of temporal trend analysis of public engagement and sentiment. This study obtained state-level COVID-19 confirmed cases data from the National Science Foundation (NSF) Spatiotemporal Innovation Center’s GitHub repository [49]. The dataset contains the cumulative number of daily confirmed cases from 22 January, 2020, to 20 May 2021, in all 50 states of the United States and the District of Columbia. The daily-confirmed-case growth rate in 48 adjoining states and the District of Columbia were derived from the dataset to compare with the public daily engagement and sentiment in this study.

### 3.5. COVID-19 Vaccination Data

On 14 December 2011, the United States started phased vaccination with the first shot administered in New York [41]. To investigate how the public opinions changed on the spatiotemporal scale as the phased vaccination progressed, this study obtained the COVID-19 vaccination number from 14 December 2020, to 20 May 2021, from a GitHub repository maintained by Johns Hopkins Centers for Civic Impact for the Coronavirus Resource Center (CRC) [50]. The datasets contain the cumulative number of daily vaccinations among the population in all 50 states, the District of Columbia, and foreign territories of the United States.

## 4. Methodology

**Figure 1:**
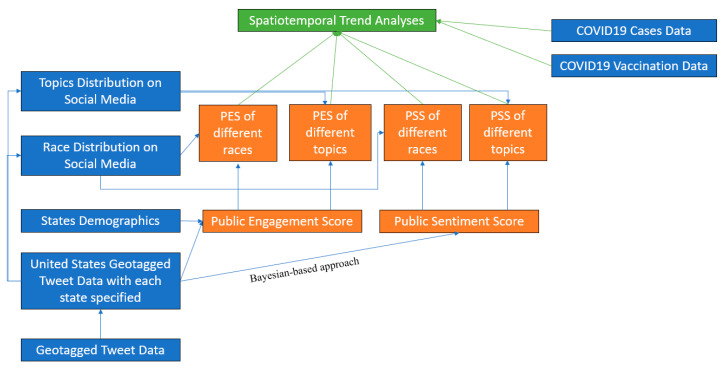
Workflow of the methods for public engagement and public sentiment.

As Figure 1 shows, this research mines public opinions from social media to understand how the public views COVID-19 vaccines by analyzing different types of data. To achieve this purpose, two social media-based measures, Public Engagement Score (PES) and Public Sentiment Score (PSS) were proposed to aid social sensing of public behaviors towards the COVID-19 vaccine. PES is to measure the level of public involvement in the discussion of vaccines, and PSS is to measure the level of public attitude toward vaccines.

### 4.1. Public Engagement Score

When the level of public involvement in the discussion of COVID-19 vaccines in different regions is measured and compared, the total volume of the discussions is highly affected by population size [22]. If the absolute volume of the discussion about the COVID-19 vaccine were the only factor considered, geographic regions with large populations would normally have a higher discussion. Such comparisons on the level of involvement are not meaningful. It is necessary to normalize the involvement by weighting the population size of geographic regions. Therefore, Public Engagement Score (PES) is proposed by the total number of tweets divided by the population size in a certain region in one day, as shown in Equation (1).

PES = N/*pop*^β^ (1)

where N is the number of vaccine-related tweets, *pop* is the state population size provided by the US Census Bureau in 2010, and β is a coefficient estimated from the relationship between tweets and population size from historical data. β is the calling exponent and ranges from 0.67 to 0.78 from prior knowledge [30]. It is set as 0.725 in this research.

### 4.2. Public Sentiment Score

Public sentiment is measured by aggregating individuals’ sentiments, which are typically classified into three sentiment polarities (i.e., positive, neutral, and negative) [25]. Textblob library with Naïve Bayes categorization model was invoked to determine the sentiment polarities. The sentiment polarity probability was determined through the following equation of Naïve Bayes.

(2) P(polarity|Features)=P(polarity)∗P(feature|polarity)/P(features)

where *P*(*features*) is the probability distribution, which is established from particular features, *P*(*polarity*) is the previous possibility of a polarity, and *P*(*feature*|*polarity*) is the previous possibility in which particular features are categorized as a polarity.

The vaccine-related tweets are only a portion of all tweets, so the aggregation of each tweet’s sentiment is not representative of the sentiment of all users on Twitter. Therefore, the daily sentiment value of available tweets can only be a probability value for the public sentiment with uncertainty. This public sentiment probability is shown in Equation (2).

(3) $$\theta_{i,r}=n_{i,r}/N_{r}$$

where θ is sentiment probability which contains three possible polarity indexes i (i.e., $\theta_{1}$ is positive, $\theta_{2}$ is neutral, and $\theta_{3}$ is negative), r is the geographic region studied, n is the number of vaccine-related tweets with a specific sentiment polarity index i, and *N* is the number of total vaccine-related tweets.

The public sentiment score (PSS) of Equation (3) is derived from Equation (2) by using the positive sentiment probability $\theta_{1\;}$ to subtract the negative sentiment probability $\theta_{3}$. It measures the daily net positive sentiment probability, and this value is used to represent baseline daily public sentiment.

(4) $$PSS=\theta_{1,r}-\theta_{3,r}$$

PSS is a subtraction of sentiment probabilities with two different polarities. It is only representative for a daily possible sentiment value with uncertainties. To address the uncertainties of public sentiment, this research adopts a Bayesian-based approach proposed by Chen [29]. The Bayesian-based approach integrates prior knowledge and newly observed evidence (i.e., social media information in this research), resulting in a reliable estimation of interest variables. Based on the availability of previous information or knowledge, the prior is created with informative or non-informative approaches. When the previous information is available, the prior is modeled as an informative prior, otherwise modeled as a non-informative prior to reflect the balance among the outcomes of interest variables. In this research, the sentiment information in the preceding week is used to model an informative prior of PSS for each date. PSS with uncertainties is measured in a daily manner during this study period (1 October 2020 to 21 May 2021).

### 4.3. Topic Analysis

**Figure 2:**
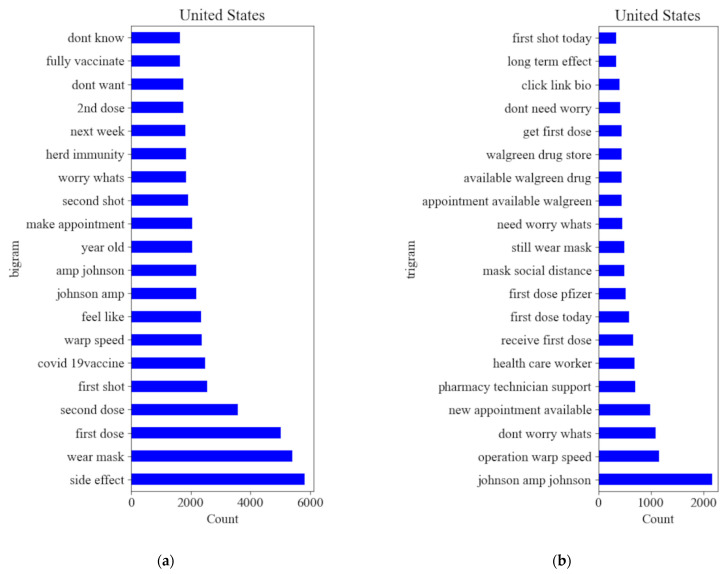
Bigrams (**a**) and Trigrams (**b**) of Keywords.

Even though this research extracts tweets by setting keywords “Vaccine” and “Vaccination,” the words “vaccine” and “vaccination” are still too vague to discover what the public has been really discussing. Public opinions might change toward different vaccine topics. For example, a person is positive about receiving the vaccine meanwhile complaining about the slow administration process of receiving the vaccine. The person expressed two opposite sentiments towards vaccines from two perspectives. Therefore, it is important to understand the PES and the PSS distribution on different topics. To observe what vaccine-related topics have been discussed and how the sentiment varies toward various aspects of vaccines, three topics were defined in this research, vaccine type, phased vaccine, and health concern. “Vaccine Type” is aiming at investigating public opinions toward several types of major vaccines, which include Pfizer/biotech, Moderna, and Johnson and Johnson. “Phased Vaccine” is aiming at investigating public opinions toward administrations related to vaccines, such as vaccine distribution and vaccine phases. “Health Concerns” is aiming at understanding public opinions on side effects and vaccine effectiveness. To approve the validity of these 3 topics, bigrams and trigrams of keywords are generated that have been most mentioned in tweets (Figure 2) and keywords are concluded for each topic in Table 2.

### 4.4. Race Analysis

Research has found that vaccines have varying effects on different races [51]. As well, various races also have different vaccine hesitancy [46]. Therefore, this study also investigated how the public opinions of various races differ from each other through social media. The spatiotemporal trend of engagement and sentiment of each race toward different topics related to vaccines were observed. As this research mentioned earlier, the race attribute for each tweet was derived by matching the last name of the user from each tweet to the last name database released by the US Census Bureau [48]. The race attribute for each tweet is randomly classified to a race in a probability with the same value as the race distribution. For instance, if a tweet was posted by a tweet user with the last name Washington with the race distribution White 5.17%, Black 87.53%, API 0.3%, AIAN 0.68%, 2PRace 3.78%, and Hispanic 2.54%, this tweet’s probability of being assigned as an African Americans was 87.5%, and same for other races. Since not all Twitter users set their user profile information formally and veritably, unpredictable tweets were classified as “unknown.” Eventually, 81,212 out of 156,207 (52%) tweets were classified for race analysis.

## 5. Result and Discussion

In this section, public engagement and public sentiment are analyzed through scales of nation, states, topics, and races. The public engagement subsection provides how much the public has participated in the discussion and how the participation has been changing during this period. Comparisons of engagement among significant states and different topics were also presented in the subsection. In addition, the change in public sentiment during the period and comparisons of sentiment among different states and different topics are introduced in the next subsection. Finally, a comprehensive public opinion study for different races toward vaccines was presented in the last subsection.

### 5.1. National Analysis

#### 5.1.1. National PES

**Figure 3:**
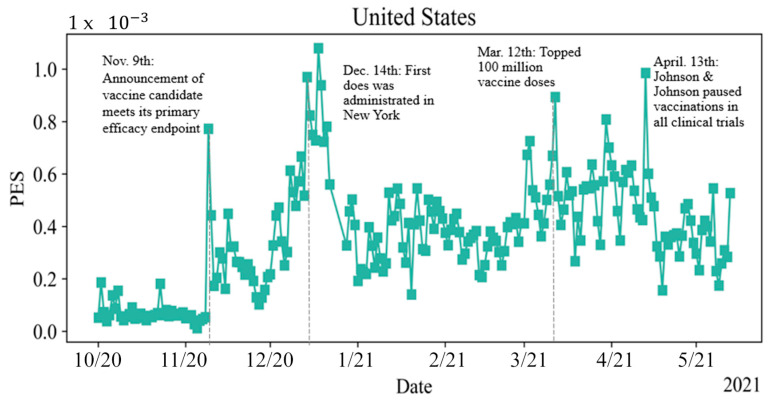
Temporal Trend of PES in the United States from 1 October 2020 to 21 May 2021.

Figure 3 describes how public engagement has been changing from 1 October 2020 to 21 May 2021. There are many spikes shown in the figure. Each spike indicates one public event related to the COVID-19 vaccine. For instance, the spikes around 9 November 2020, are due to the announcement that the vaccine candidate meets its primary efficacy endpoint. The spike on Dec. 14 is due to the first vaccine dose in New York. The high volume of discussion on 2 March is because President Donald Trump was exposed by news media that he and his family secretly got vaccines before leaving The White House. The spikes around 12 March are because of the blood clots caused by the AstraZeneca vaccine, remarks of the American Rescue Plan, and the administration of 100 million vaccines. The last spike on 13 April is because Johnson and Johnson paused its vaccinations in all clinical trials. The public who took its AstraZeneca vaccine were very panicking and had a high volume of discussions.

#### 5.1.2. National PSS

**Figure 4:**
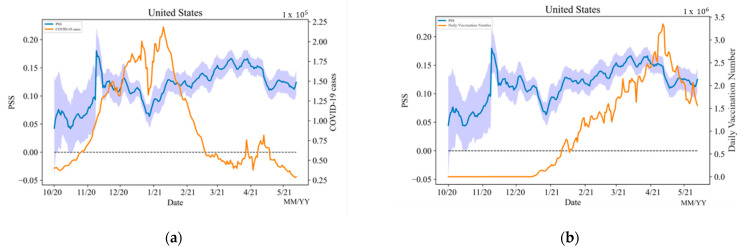
(**a**) Temporal Trend of daily PSS vs. COVID-19 Daily Case number, (**b**) Temporal Trend of daily PSS vs. Daily Vaccination Number in the United States from 1 October 2020 to 21 May 2021.

The blue line in Figure 4a shows the temporal trend of daily PSS and the border around it shows the uncertainty value of the PSS each day. The orange line shows the COVID-19 daily case number. The blue line in Figure 4b also shows the temporal trend of daily PSS but the orange line shows the daily vaccination number. Although the PSS are changing temporally, generally PSS toward vaccines is positive. It shows that the majority of people in the United States believe that vaccines can help resolve this pandemic. Before the first vaccine was announced to meet its primary efficacy endpoint, the public sentiment was relatively low. This is because people had not known when the vaccine would be available. In addition, during the first period, the number of vaccine-related tweets was relatively low, therefore, the uncertainty level varies more than in the rest of the periods. On 9 November 2020, the date of the first vaccine was announced to meet its primary efficacy endpoint, and the PSS increases tremendously. It shows that the public was very positive when hearing this news. However, at that time the United States was experiencing the most challenging time in this pandemic. The daily confirmed COVID-19 cases had reached the highest point, meanwhile, the federal government was just starting the vaccine distribution. Therefore, between the first successful vaccine announcement and late December, PSS went down. When the year 2021 started, PSS had an increasing trend. This is because the number of COVID-19 daily confirmed cases had dropped, and the vaccination rate of the whole nation had been increasing over time. In mid-April 2021, the PSS dropped down due to the increase of the daily confirmed cases and the decrease in the daily vaccination number.

### 5.2. State Analysis

**Figure 5:**
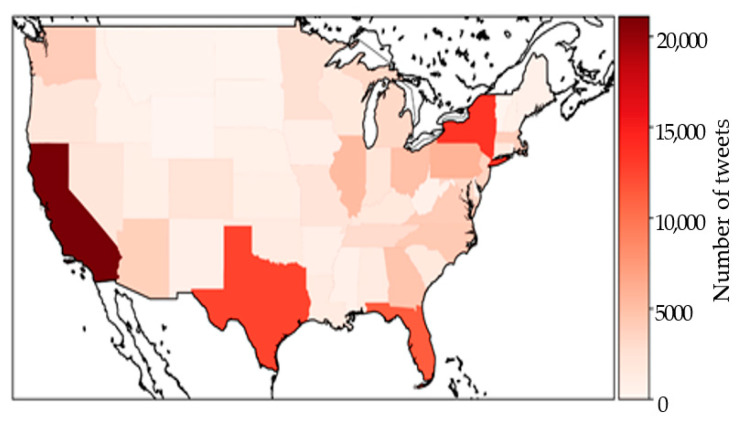
Number of tweets in the United States from 1 October 2020 to 21 May 2021.

Figure 5 demonstrates the spatial distribution of geotagged tweets posted during the study period; the four states, California (CA), Texas (TX), New York (NY), and Florida (FL) have the highest number of tweets posted. The major reason is that CA, NY, TX, and FL have the highest population, number of tweets, and number of vaccinations [34]. As mentioned earlier, fewer tweet posts bring more uncertainty to the sentiment probabilities. Therefore, this study compared the PSE of all states but uses CA, NY, TX, and FL as examples to compare the PSS pattern for states.

#### 5.2.1. State PES

**Figure 6:**
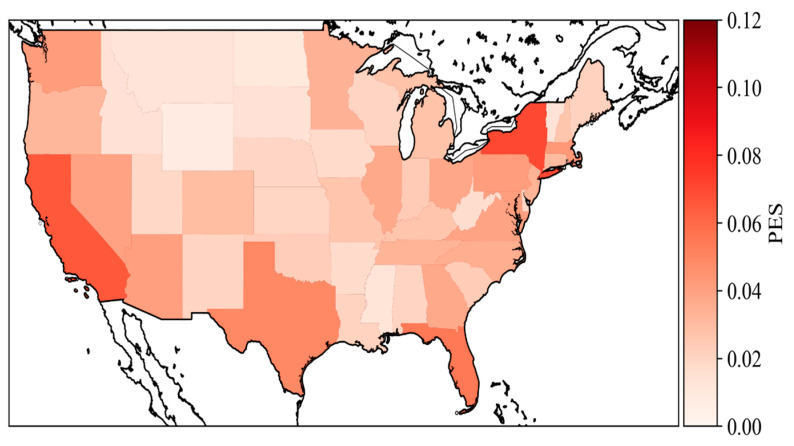
Spatial distribution of PES in the United States from 1 October 2020 to 21 May 2021.

**Figure 7:**
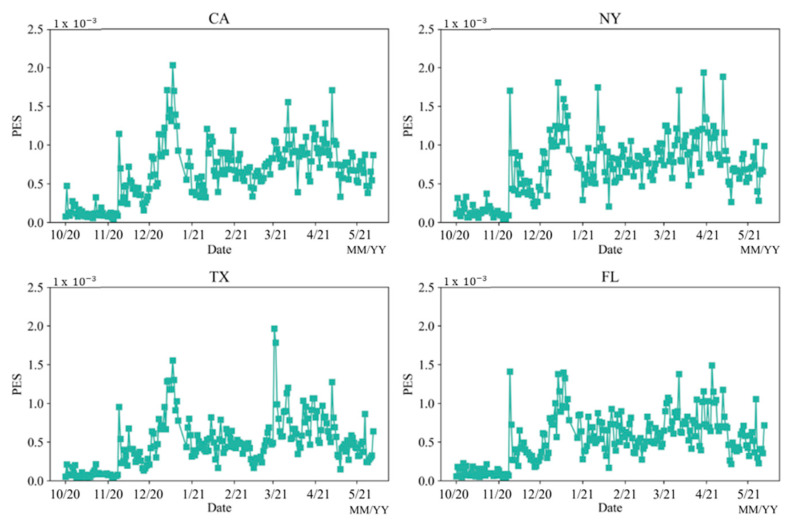
Temporal patterns of PES in the selected four states from 1 October 2020 to 21 May 2021.

Due to the high PES of those four states mentioned above, this study focused on the PES in these four states for Comparative analysis. Figure 6 shows the spatial distribution of PES in each state, but Figure 7 only shows the temporal patterns of PES in the selected four states. The selected four states are very similar to the national trend. New York had more spikes compared to the other three states. This is likely because there were frequent COVID policy changes and more COVID news reports compared to the other states [52,53]. Florida had fewer PES compared to the other three states. This is likely because the Florida government has always opposed strict control policies, such as mask mandate and vaccine mandate policies [54,55]. It can affect the involvement of public discussion. In addition, Texas had a large spike in early March compared to other three states. This is because, at that time, Texas governor Greg Abbott lifted the mask mandate and fully reopened Texas on 2 March while many publics did not even receive one dose of vaccines yet [56].

#### 5.2.2. State PSS

**Figure 8:**
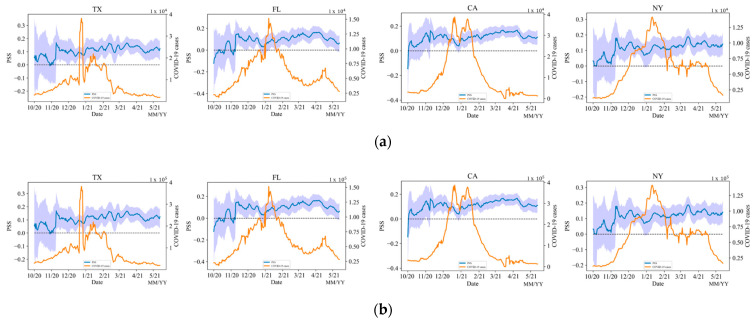
(**a**) Temporal Trend of daily PSS vs. COVID-19 Daily Case Numbers, (**b**) Temporal Trend of daily PSS vs. Daily Vaccination Numbers in the selected states.

Figure 8 shows that the states have similar patterns as the whole nation. When the first successful vaccine was announced, the PSS trend increased. Meanwhile, as the COVID-19 case number increased, the sentiment dropped down toward the end of the year 2020. When the daily vaccination number increased, the sentiment also increased. Most of the time, the public is positive towards vaccines. Different states have different times with high spikes of COVID-19 cases, which affects the trend of public sentiment before vaccines were largely distributed. The PSS of Florida is relatively lower than the other three states. Again, this is also likely because the Florida government has always tried to prohibit vaccine mandate policies in schools and businesses [54,55].

### 5.3. Selected Topics Analysis

Various people engaged in vaccine discussions from different perspectives. In addition, during different periods of experiencing the COVID-19 vaccine, people may have varying focus and opinions in their discussion. Public opinions were observed in detail by analyzing the engagement distribution on different topics in different time periods.

In the temporal trend of nation PES, there are four spikes in Figure 3. Therefore, the study period was divided into four sub-periods, the first sub-period is the time before the announcement that the first vaccine candidate meets its primary efficacy endpoint. This sub-period represents when most of the public has not known when the vaccine would be available. The second sub-period is between the date of the announcement and the very first dose of the vaccine. This sub-period represents when the vaccines had been available, but the vaccination process had not yet begun. The third sub-period is between the date of the very first dose and the date of reaching 100 million vaccines administered. This sub-period represents when the public started the vaccination process. The last sub-period is after the date of reaching 100 million vaccines administered. This sub-period is a milestone that represents the time many people had been vaccinated. This study utilized a boxplot to demonstrate PES distribution by presenting its median, first quartile, and the third quartile for different topics during each period.

#### 5.3.1. PES Analysis

**Figure 9:**
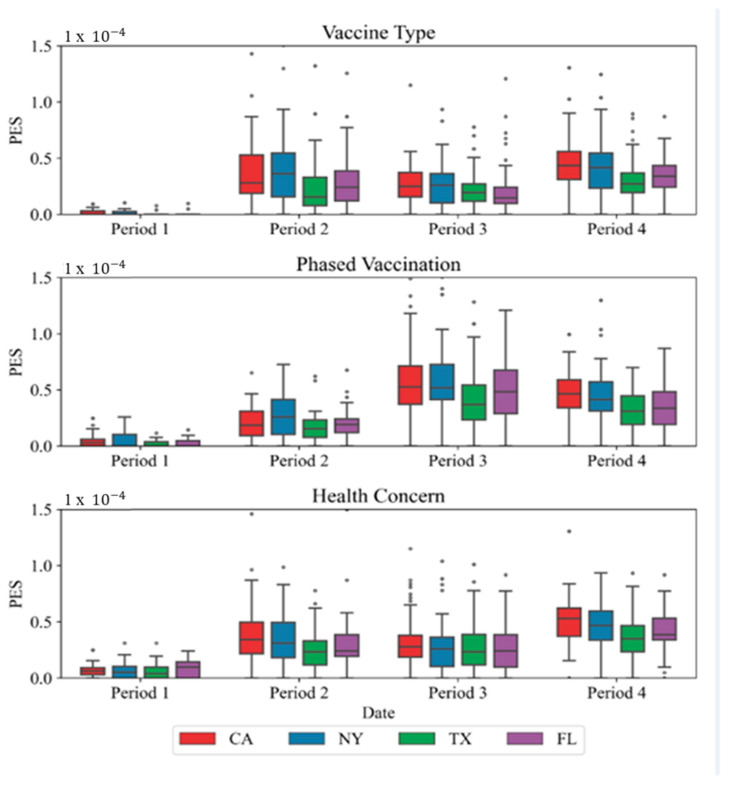
PES changes over time on different topics in the selected four states. The * symbol in the figure represents the noise of the data.

As Figure 9 shows, in period 1, the public had barely heard about vaccines, especially the name of vaccine brands, so the public has less discussion compared to other 3 time periods, but the public discussed more about vaccine administrations and their health concerns toward vaccines compared to discussion on vaccine types. In period 2, the discussion in three aspects all increased, because vaccine approvals were gradually announced, and many people had been waiting for the vaccine to end this pandemic. During this period, vaccine types had more discussions than two other aspects. In period 3, the PES of Phased Vaccination increased significantly over the previous two periods as the public started to wonder when and how they can receive vaccines. Many publics were willing to take vaccines, but they were not the first priorities who can take the vaccines. The public complained a lot about administration policies during this period. In period 4, the public discussed more health concern topics and vaccine types. As the CDC guideline indicates, people who have been vaccinated might receive side effects such as pain, redness, and swelling in the arm where they received the shot, as well as other side effects throughout the rest of the body [57]. In period 4 when many people got the second dose of the vaccine, most people got some of the side effects listed above. Therefore, in period 4, more people started to compare different vaccine types and have more discussions about their health conditions. During this period, more than 50% of the population at least got 1 dose of any type of vaccines [44], and most of the public knew how and where to obtain the vaccines. Therefore, the discussion about phased vaccination was reduced in period 4.

#### 5.3.2. PSS Analysis

**Figure 10:**
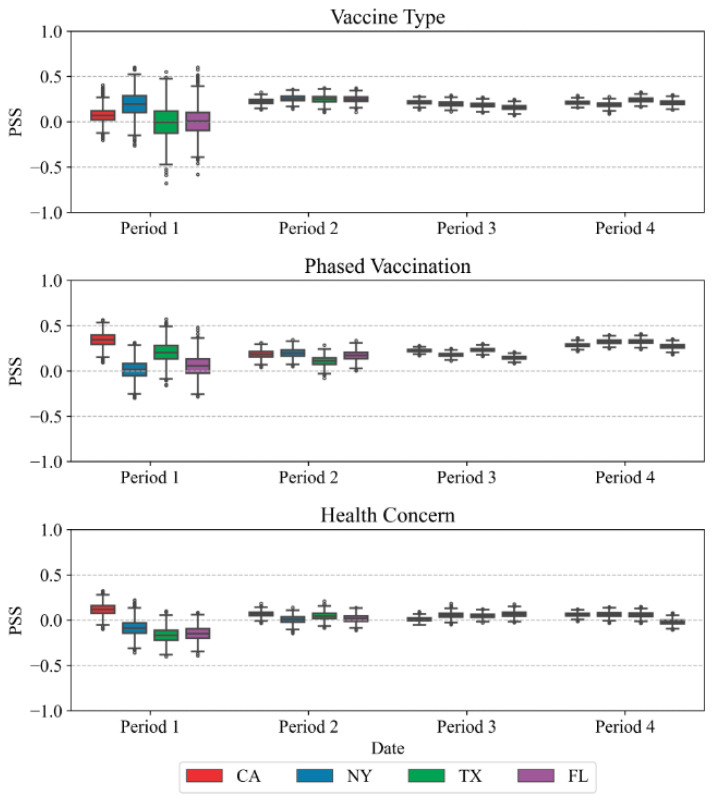
PSS changes over time on different topics in the selected four states.

In this section, to avoid the extreme value of PSS on particular days, PSS for each topic has been averaged by week. Figure 10 shows the maximum, the first quartile, the median, the third quartile, and the minimum of weekly PSS values. In period 1, the total tweets about vaccines were relatively low, which caused large sentiment variations. In the rest of the periods, the PSS was more stable. The public had a more positive attitude when discussing vaccine types and phased vaccination, but they had a lower sentiment when discussing their health concerns. This is because when the public discussed topics such as side effects, they were more worried. Negative attitudes are normally expressed in such a context. PSS on phased vaccination has increased significantly from period 3 to period 4. That means the public became more satisfied with the administration of the vaccination process. In period 3, people had concerns about when they could become vaccinated. Many people complained they could not obtain the vaccine during that period. In period 4, an increasing number of people who wanted to receive the vaccination had been vaccinated, so there is an increase in PSS on phased vaccination. In addition, State Florida has a significant drop in health concerns. This shows after Johnson & Johnson’s AstraZeneca vaccine was exposed for causing blood clots and it paused vaccinations in all clinical trials, the public had more concerns about the side effects of vaccines.

### 5.4. Race Distribution Analysis

The federal policy defines “Hispanic” not as a race, but as an ethnicity [58]. The authors understand that there are subtle differences between the two terms. A survey finds that two-thirds of Hispanics believe their Hispanic background is a part of their racial background but not something separate [58], and in the last-name database released by the U.S. Census Bureau in 2010 also used Hispanic as a race. Therefore, the vocabulary “race” is used interchangeably with “ethnicity”.

#### 5.4.1. PES

**Figure 11:**
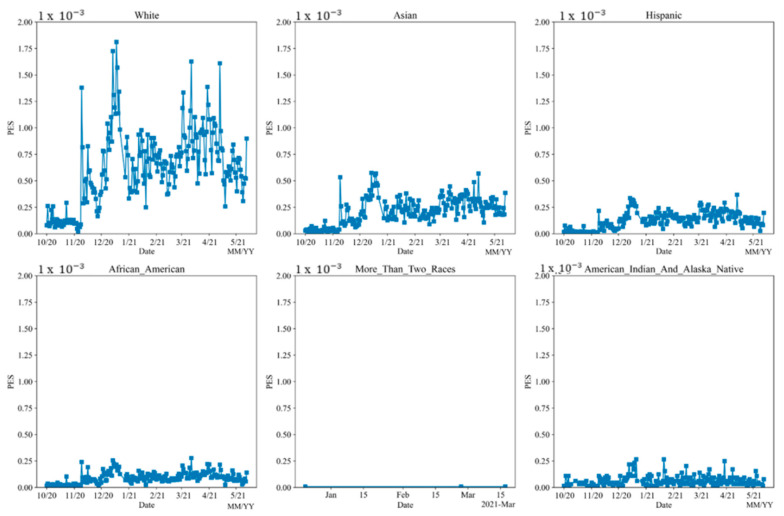
PES analysis on different races from 1 October 2020 to 21 May 2021.

Figure 11 shows that different races have varying PES. When the PES for each state was calculated, the PES value was normalized by the population size of each state. In race analysis, the PES value for different races was normalized by the race population. All races follow a similar pattern to the national PES pattern. In addition, whites have the highest engagement through all times, followed by Asians, Hispanics, African Americans, American Indians, Alaska Natives, and more than 2 races. The absolute number of tweets for American Indians is low, but from the graph below, American Indians have a relatively high engagement considering their small population size. Smaller numbers of tweets cause a larger variation of sentiment, which leads to inaccurate sentiment results. Therefore, more than 2 races and American Indians are not included in the subsequent discussions.

#### 5.4.2. PSS

**Figure 12:**
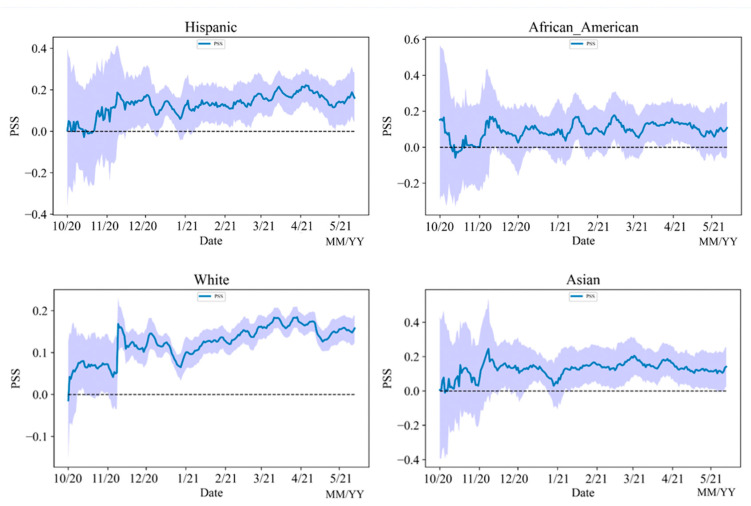
PSS analysis on different races from 1 October 2020 to 21 May 2021.

Figure 12 shows that in the first period, the number of vaccine-related tweets was relatively low. Therefore, the uncertainty levels of sentiment during the first period were high among all four races. From the second period to the fourth period, the uncertainty level of whites varies much less than the other three races, because the number of tweets from whites is much higher than other races. Whites, Asians, and Hispanics have similar PSS patterns as the national PSS trend, but the PSS pattern of African Americans did not follow the national PSS trend, which might indicate that African Americans’ attitudes toward vaccines are not as positive as the other three races. The reasons leading to this result might be of the history of medical abuse African Americans endured. Being the subject of medical testing in the past caused many African Americans to distrust the COVID-19 vaccines [59]. Meanwhile, African Americans have the highest death rate of COVID-19 among all races [60], which makes them show more negative attitudes in their social media posts.

## 6. Conclusions

This research analyzes public opinions toward vaccines through social media from the following three perspectives: (1) How the public engagement and public sentiment toward vaccines change spatiotemporally; (2) How the public reacts toward different topics related to vaccines; (3) How each race differs in their social media engagement and sentiment expressed toward vaccines.

This research adopted existing metrics to measure the public engagement and public sentiment. Specifically, PES is used to measure the public involvement level of vaccine-related discussions in a certain region, and PSS is used to measure the public attitude toward vaccine-related discussions. This research investigated the spatiotemporal trend of PES and PSS on both national scale and state scales. Results show that PES is intensely related to social events. Before the year 2021, PSS was inversely correlated with the number of newly confirmed COVID-19 cases, since the vaccines had not been widely administered. However, the PSS was correlated with the number of newly vaccinated people since the beginning of 2021. It shows that the public was more positive when more people got vaccinated.

PES and PSS analyses are also conducted from different perspectives, including various topics, three brands of vaccines, and different races. This research found that when an increasing number of people got the vaccine, the public was more satisfied with the administration. After the Johnson & Johnson vaccine was exposed for causing blood clots, the public had more discussions about vaccine types and health concerns. As a result, PSS toward Johnson & Johnson dropped. Race inequality was also revealed through PES and PSS. Whites have a much higher PES and a more stable PSS than minorities. African Americans have a very unstable sentiment toward vaccines.

This research has several limitations. First, it is limited by a smaller number of tweets, especially in certain geographic regions and demographic groups. The PES and PSS of “More than two races” and “American Indians and Alaska Natives” were not analyzed due to their small number of geotagged tweets. One of the main reasons is because this research only employs geotagged tweets which only account for around 1%–2% of all tweets. Furthermore, previous studies have found that Twitter users tend to live in populous counties and sparsely populated counties are significantly underrepresented [61]. Urban users are over-represented and provide more information than rural users [62]. Therefore, the sentiment results might not show the public attitude in regions with low populations. In addition, another study showed that messages with images have a higher rate of liking and sharing than messages without images. This study only analyzed the text information related to COVID-19 vaccines but did not analyze related images posted on Twitter. Data could be richer if such images were also included. Lastly, social media users tend to be young and more educated compared to the general population [63], so the tweet may introduce elements of bias into the data, which can cause the inaccuracy of engagement and sentiment results.

Our research provides reliable spatiotemporal results showing public engagement and public sentiment during the vaccination phases. It can be utilized for future spatiotemporal research and inequality research related to vaccines. In the future, tweets with no geotag could be added and Name Entity Recognition (NER), a method manually analyzing the geolocation entity of a tweet based on its content or without considering the geographical area, could be deployed to enrich data sources and produce a more comprehensive analysis on the public engagement and public sentiment. The methodology described in this study is also applicable for understanding the public engagement and sentiment on other events in other spatiotemporal extends, if there are a significant number of social media users for the chosen topic, such as natural disasters, war, and conflicts, economic crisis, policy debates, elections, etc.

## Figures and Tables

**Table 1:** An example of race distribution of the last name from the US Census Bureau in 2010.

Name	White	Black	API	AIAN	2Prace	Hispanic
Smith	70.9	23.11	0.5	0.89	2.19	2.4
Washington	5.17	87.53	0.3	0.68	3.78	2.54
Chen	1.4	0.3	96.12	0.02	1.64	0.52
Beyale	1.89	0	0	94.7	0	1.89
KANEKOA	10.28	0	25.23	0	58.88	5.61
CEBALLOS	3.97	0.33	1.13	0.24	0.32	94.01

**Table 2:** Vaccine-related aspects and their related keywords.

Aspect	Keywords
Vaccine type	Pfizer, Moderna, Johnson amp Johnson, Janssen, biotech
Phased Vaccination	frontline, phased, first dose, healthcare, old, second dose, operation, registration, CVS, pharmacy, administration, essential, medical condition, front line, health care
Health concern	side effect, mask, die, warp speed, warp, fever, tiredness. headache, muscle pain, fever, chills

## Data Availability

Data is not in public domain for privacy issue, and maybe available for research purposes if contact authors.

## Abbreviations

To avoid confusion regarding the usage of abbreviations in this article, all abbreviations used in this article are listed in the following definition tables.

Abbreviations	Definition
CDC	Centers for Disease Control and Prevention
CGA	The Harvard Center for Geographic Analysis
COVID-19	an infectious disease caused by the SARS-CoV-2 virus
FDA	U.S. Food and Drug Administration
HCW	Health Care Workers
NER	Name Entity Recognition
PES	Public Engagement Score: a score shows the level of public involvement for discussion of COVID-19 vaccines
PSS	Public Sentiment Score: a score shows the polarity of public attitude for discussion of COVID-19 vaccines
